# Effects of coral colony morphology on turbulent flow dynamics

**DOI:** 10.1371/journal.pone.0225676

**Published:** 2020-10-07

**Authors:** Md Monir Hossain, Anne E. Staples

**Affiliations:** Engineering Mechanics Program, Department of Biomedical Engineering and Mechanics, Virginia Tech, Blacksburg, VA, United States of America; Coastal Carolina University, UNITED STATES

## Abstract

Local flow dynamics play a central role in physiological processes like respiration and nutrient uptake in coral reefs. Despite the importance of corals as hosts to a quarter of all marine life, and the pervasive threats facing corals, characterizing the hydrodynamics between the branches of scleractinian corals has remained a significant challenge. Here, we investigate the effects of colony branch density and surface structure on the local flow field using three-dimensional immersed boundary, large-eddy simulations for four different colony geometries under unidirectional oncoming flow conditions. The first two colonies were from the *Pocillopora* genus, one with a densely branched geometry, and one with a comparatively loosely branched geometry. The second pair of geometries were derived from a scan of a single *Montipora capitata* colony, one with the roughness elements called verrucae covering the surface intact, and one with the verrucae removed. For the *Pocillopora* corals, we found that the mean velocity profiles changed substantially in the center of the dense colony, becoming significantly reduced at middle heights where flow penetration was poor, while the mean velocity profiles in the loosely branched colony remained similar in character from the front to the back of the colony. For the *Montipora* corals, somewhat counterintuitively, the colony without verrucae produced almost double the maximum Reynolds stress magnitude above the colony compared to the colony without verrucae. This implies that the smooth colony will have enhanced mass transport and higher bed shear stress and friction velocity values relative to the colony with verrucae.

## Introduction

A new and important direction in understanding the coupled dynamics of corals and their hydrodynamic environments seeks to measure and model the flow inside coral reefs and individual colonies. But the task is challenging due to the existence of a wide range of flow scales within different boundary layers around a coral reef [1, 2]. The transport of planktonic food in the reef is mainly governed by the large-scale flow motion, while diffusion [3, 4] takes place at the surface of the coral at a much smaller scale. Experimental studies performed at this smaller hydrodynamic scale [5, 6] show that the growth direction, dimensions, and sparsity of branches depend on the flow profile inside the coral [7–9]. Similarly, the velocity profile controls the nutrient distribution [7, 10–13]. and physiological processes like photosynthesis and respiration [14–17] near the coral surface. Flow motion also controls the thermal micro-environment [18, 19] at the coral surface, which is extremely important for phenomena like coral bleaching. Both the transfer of nutrients from the overlying water column to the colony and the transport of gases from the coral to the water column depend on the detailed flow dynamics through and around the coral [20, 21]. All these physiological activities are directly related to the flow conditions in and around the coral. To obtain a clearer characterization of these local flow fields, the authors tried to find answers to a few basic questions, including, how does the mean flow profile change with branch density in and above a single coral colony? And, what are the effects of surface roughness on the mean flow profile and the local turbulence characteristics?

Numerous prior studies have been performed on coral reefs and above the canopy [22, 23], but these analyses have largely lacked measurements of the detailed flow field inside the coral because of the complexity in measuring the flow variables at the interior of a colony or reef or due to a lack of optical and acoustic access. Though experimental flow field measurements have been performed on corals, data obtained at several points are not sufficient to measure the complete flow profile inside a coral. Canopy flow theory may be suitable to model the detailed flow dynamics inside a coral reef. In canopy flow studies on corals by Shavit *et al*., the model reef geometries were composed of homogeneous, simplified elements and the double averaged Navier-Stokes equations [24] were used to simplify the analysis, which led to the modeling of a new unresolved dispersive stress. [25, 26]. To mimic the hydrodynamics at the interior of a coral reef, Lowe *et al*. [27] used an array of cylinders to capture the velocity profile and shear stress inside the reef. This model was also used to estimate the mass transfer from the surface of the coral. Bilger and Atkinson [28] used engineering mass transfer [29] correlations to predict rates of phosphate uptake by coral colonies and treated the top of the colonies as a rough boundary. Recently, Stocking *et al*. examined the impact of surface roughness on turbulent hydrodynamics and heat transfer above a grooved brain coral, which because of its convex spherical shape, provides excellent optical and acoustic access [30]. Chang *et al*. [31] used magnetic resonance velocimetry [32] to reveal the internal flow profile in two coral colonies with different branching patterns. The results provided an excellent visualization of the flow field inside the corals, but repeating such experiments on diverse coral structures in order to characterize how the mean velocity profile and other flow quantities differ for different geometries is expensive and unrealistic. To alleviate this problem, numerical simulations can be an important tool.

For numerical simulations, meshing around the arbitrary branches of a complex coral geometry is a challenging task. For a body-fitted mesh, even the simplest geometries require efficient meshing algorithms to reduce the computational cost. To overcome this issue, numerical experiments in many previous studies were performed by considering coral as an isotropic, porous medium, but questions about accuracty emerge due to this vast simplification [33]. Kaandorp *et al*. used the Lattice Boltzmann method to understand the effects of flow on a realistic coral structure and simulated flow patterns around the coral surface [34, 35], but was limited to low Reynolds numbers (154 to 3,840), due to stability issues. Similarly, Chindapol *et al*. [36] used COMSOL Multiphysics to analyze the effects of the surrounding flow on coral growth, but were limited to laminar flow studies because of the computational cost. Chang *et al*. [37] were the first, according to the authors’ knowledge, to use the immersed boundary method with large-eddy simulations to compute the flow through and around a densely branched coral colony geometry. Their analysis was mainly focused on local shear and mass transfer on the coral structure. Recently Osorio-Cano *et al*. computed the colony drag coefficient for the loosely branched coral *Acropora palmata* [38]. In another recent paper, Hossain and Staples *et al*. [39] computed detailed velocity fields and mean flow profiles in the interior of a *Pocillopora* colony, and showed that the mass transfer rate remains almost constant throughout the coral even when the mean velocity drops substantially inside it. In this work, we calculate the velocity fields and mean velocity profiles in two different *Pocillopora* branching colonies with significantly different branch densities and report how the mean flow profiles differ in these loosely and densely branched colonies.

In addition to characterizing the mean velocity profiles for different colony branching patterns, it is important to understand the effects of natural flow conditions on coral morphology and vice versa. To flourish in a changing environment, corals modify their structure to adjust to the surrounding hydrodynamics. It has been found that under sheltered flow conditions, *Pocillopora damicornis* and *Seriatopora hystrix*, transform from thick and densely-branched colony geometries to thin with greater branch spacing geometries due to the difference in flow conditions [40]. Kaandorp and Kubler reported similar transformations for *Madracis mirabilis* [41]. Even a small modification in the flow environment can affect the boundary layer and hence the turbulent stresses developed at the coral surface, and can change the mass transfer rates significantly [42–44]. These variations in turbulent flow profiles illustrate the effects of coral structure on hydrodynamics in different flow conditions. One such interaction can be observed in the hydrodynamics of the loosely branched coral *Montipora capitata* in a strong flow environment. Normally, loosely branched coral grows in quiet flow environments, but *Montipora capitata*, which has roughness elements called verrucae on its surface, is found in turbulent flow conditions near Hawaii. Naturally, questions arise regarding the impact of the verrucae on the surrounding flow. To investigate the influence of the surface structure on the surrounding flow, we compared the boundary layer and turbulent stresses on nearly identical *Montipora capitata* colonies, one with, and one without verrucae.

In light of the discussion above, the current manuscript can be divided into two main parts. In the first part, three-dimensional velocity field components and mean velocity profiles were computed at the interior of two *Pocillopora* corals with different branching patterns and densities for the same oncoming flow conditions. In the second part of the manuscript, a comparative study was performed between velocity profiles and turbulent stresses developed on nearly identical *Montipora capitata* colonies, one with, and one without verrucae, at two different Reynolds numbers, which correspond to natural low and high flow conditions, respectively.

## Materials and methods

### Coral species and geometries

**Fig 1:**
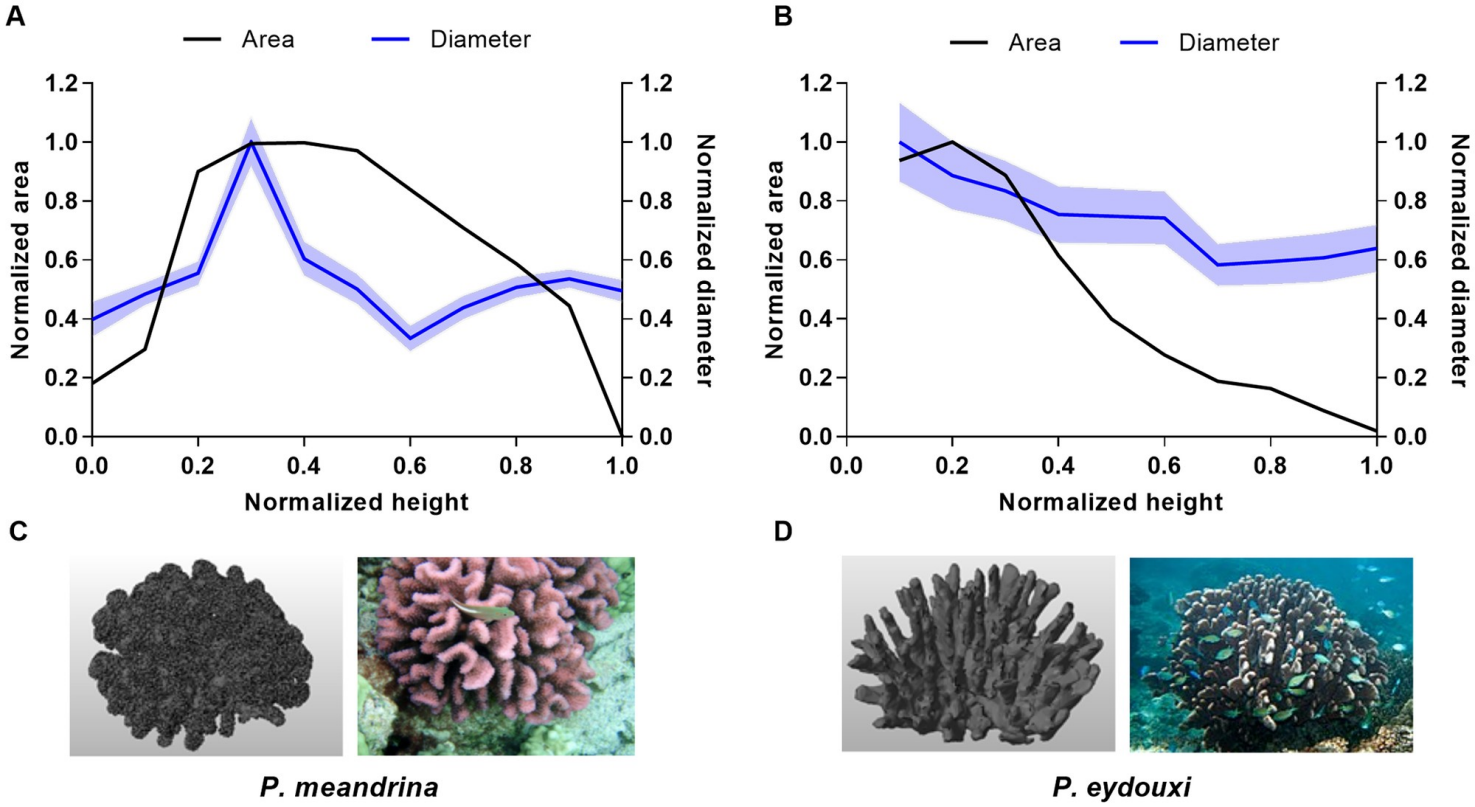
*Pocillopora* colony geometries used in the current simulations. (A) Variation of cross-sectional area and branch diameter versus height for *P. meandrina*. (B) Variation of cross-sectional area and branch diameter versus height for *P. eydouxi*. The mean diameter was obtained by averaging the branch diameters at each height. (C) Rendered stereolithography file of the *P. meandrina* colony used in the simulations and a *P. meandrina* colony in its natural environment on the ocean floor. [Brocken Inaglory, *P. meandrina* with a resident fish, Wikimedia Commons (2008); used in accordance with the Creative Commons Attribution (CC BY-SA 4.0) license [45]. (D) STL file of the *P. eydouxi* colony and a *P. eydouxi* colony in its natural environment. [Paul Asman and Jill Lenoble, around antler coral *P. eydouxi*, Wikimedia Commons (2012); used in accordance with the Creative Commons Attribution (CC BY-SA 2.0) license [46].

Stereolithography (STL) files of the coral geometries used in the current studies are shown in Fig 1. The comparatively densely branched *P. meandrina* was obtained from a computed tomography (CT) scan of a real coral skeleton from Professor Uri Shavit’s laboratory in the Department of Civil and Environmental Engineering at the Technion—Israel Institute of Technology. *P. meandrina*, commonly known as cauliflower coral, is common in the East Pacific and the Indo-West Pacific, and is mainly found on the exposed front of reefs. The stream-wise length, width and height of *P. meandrina* used in the current simulation were 0.172 m, 0.172 m and 0.10 m respectively. The branches in the *P. eydouxi* colony shown in Fig 1 are more sparse compared to the *P. meandrina* colony. This coral is common throughout the Indo-West Pacific and occurs in most reef environments, especially at reef fronts where the currents are strong. The length, width and height of *P. eydouxi* used in the simulation were 0.12 m, 0.11 m and 0.10 m respectively. Though the genus is the same, there are substantial structural differences between these two coral morphologies. The ratio of surface area of *P. meandrina* and *P. eydouxi* was 5:1 and the value of volume of bounding box (*L* × *W* × *H*) to surface area was approximately 2.61 times higher for *P. eydouxi* in comparison to *P. meandrina*. The inter branch distance to branch diamter ratio for *P. eydouxi* were double for the same value for the *P. meandrina*, which indicates that the branches were more compact inside *P. meandrina* than *P. eydouxi*. The ratio of maximum projected area in the flow direction to surface area were 0.0754 and 0.0556 for *P. meandrina* and *P. eydouxi*, respectively, which represents a higher projected surface area for *P. meandrina*. To further understand their structural differences, the variation of the mean diameter of the individual branches inside the corals and the cross-sectional areas of the colonies in the *x*-*y* plane were plotted in Fig 1 as a function of height for both *Pocillopora* colonies. The surface area is directly measured from the geometry of the STL file by CAD software.

To understand the effects of verrucae on the surrounding fluid, simulations were performed for two nearly identical coral geometries. One with, and one without verrucae. Both coral geometries originated from the same *Montipora capitata* stereolithography (STL) geometry file. In one of the *M. capitata* colonies, the verrucae were completely removed from the coral surface while keeping the dimensions of the geometry the same. The length, width and height of the corals were 0.118 m, 0.114 m and 0.1 m respectively. The cone-shaped verrucae had a base diameter of approximately 3 mm and a length of 3-4 mm. *M. capitata* is usually found in highly turbulent flow conditions in the tropical north and central Pacific Ocean near Hawaii at approximately 20 m depth. All coral species in the current study have low living-tissue biomass, so using skeletons instead of coral with bio-tissue had a minimal effect on the resulting flow fields.

### Numerical methodology

The immersed boundary method (IBM) can be implemented easily for complex geometries while minimizing computational costs. In IBM, the computation is performed on a Cartesian grid and the interface is tracked by identifying solid and fluid elements in the flow domain. For the current analysis, the IBM was implemented in a large eddy simulation (LES) framework for a turbulent flow field where the largest scales of motion were calculated and the effects of the small scales were modeled. A top-hat filter was applied implicitly to the Navier-Stokes equations by the finite-difference operators and the resulting filtered equations are as follows:

$$\begin{array}{c} \frac{\partial {\bar{u}}_{i}}{\partial t}+\frac{\partial {\bar{u}}_{i}{\bar{u}}_{j}}{\partial x_{j}}=-\frac{1}{\rho }\frac{\partial \bar{P}}{\partial x_{i}}+\nu \frac{\partial^{2}{\bar{u}}_{i}}{\partial x_{j}x_{j}}-\frac{\partial \tau_{ij}}{\partial x_{j}}+f_{i} \end{array}$$ (1)

$$\begin{array}{c} \frac{\partial {\bar{u}}_{i}}{\partial x_{i}}=0 \end{array}$$ (2)

where ${\bar{u}}_{i}$ represents the large-scale velocity vector, *P* represents pressure, *ρ* represents density, *ν* represents the kinematic viscosity, and *f*_*i*_ represents the external body force used to implement the boundary conditions for arbitrary geometries with a non-conforming grid. Here, the overbar denotes a filtered variable.

Performing the spatial filtering operation on the Navier-Stokes equations produces the subgrid scale stress term, $\tau_{ij}=\bar{u_{i}}\bar{u_{j}}-{\bar{u}}_{i}{\bar{u}}_{j}$. Now, if we use $u=\bar{u}+u^{\prime }$ in the subgrid stress tensor, where *u*′ is the residual from the filter, new terms will be generated which cannot be evaluated directly. So the *τ*_*ij*_ term needs a closure model to solve the system of Eqs (1) and (2). The dynamic eddy-viscosity model used in this study is of the following form:

$$\begin{array}{c} \tau_{ij}-\frac{\delta_{ij}}{3}\tau_{kk}=-2C{\bar{\Delta }}^{2}|\bar{S}|{\bar{S}}_{ij} \end{array}$$ (3)

where $|\bar{S}|$ is the magnitude of the large-scale strain rate tensor, *δ* is the Kronecker delta, ${\bar{S}}_{ij}$ is the strain tensor and $\bar{\Delta }$ is the filter size, which can be defined as:

$$\begin{array}{c} {\bar{S}}_{ij}=\frac{1}{2}\left(\frac{\partial {\bar{u}}_{i}}{\partial x_{j}}+\frac{\partial {\bar{u}}_{j}}{\partial x_{i}}\right) \end{array}$$ (4)

$$\begin{array}{c} \bar{\Delta }={\left(\Delta x\Delta y\Delta z\right)}^{\frac{1}{3}}. \end{array}$$ (5)

Here, Δ*x*, Δ*y* and Δ*z* are the local grid size, and C is the user-specified value for the Smagorinsky eddy viscosity model, where the usual value of C ranges from 0.1 to 0.2. However, this model does not behave properly near solid boundaries. So, in the computations, the Lagrangian averaging procedure was used to compute the value of C using information of the resolved scales [47]:

$$\begin{array}{c} C=-\frac{1}{2}\frac{L_{ij}M_{ij}}{M_{ij}M_{ij}} \end{array}$$ (6)

where

$$\begin{array}{c} {Ł}_{ij}=\hat{{\bar{u}}_{i}{\bar{u}}_{j}}-\hat{{\bar{u}}_{i}}\hat{{\bar{u}}_{i}} \end{array}$$ (7)

$$\begin{array}{c} M_{ij}={\hat{\bar{\Delta }}}^{2}|\hat{\bar{S}}|\hat{{\bar{S}}_{ij}}-{\bar{\Delta }}^{2}|\bar{S}|{\bar{S}}_{ij}. \end{array}$$ (8)

An additional test filter, $\hat{\bar{\Delta }}$, which was twice the size of the grid filter, $\bar{\Delta }$, was used to compute the value of *C*. All of the simulations in this study were performed using the IBM implemented in the LES framework using a code developed by Balaras *et al*. The complete details of the numerical method can be found in Balaras *et al*. [48].

**Fig 2:**
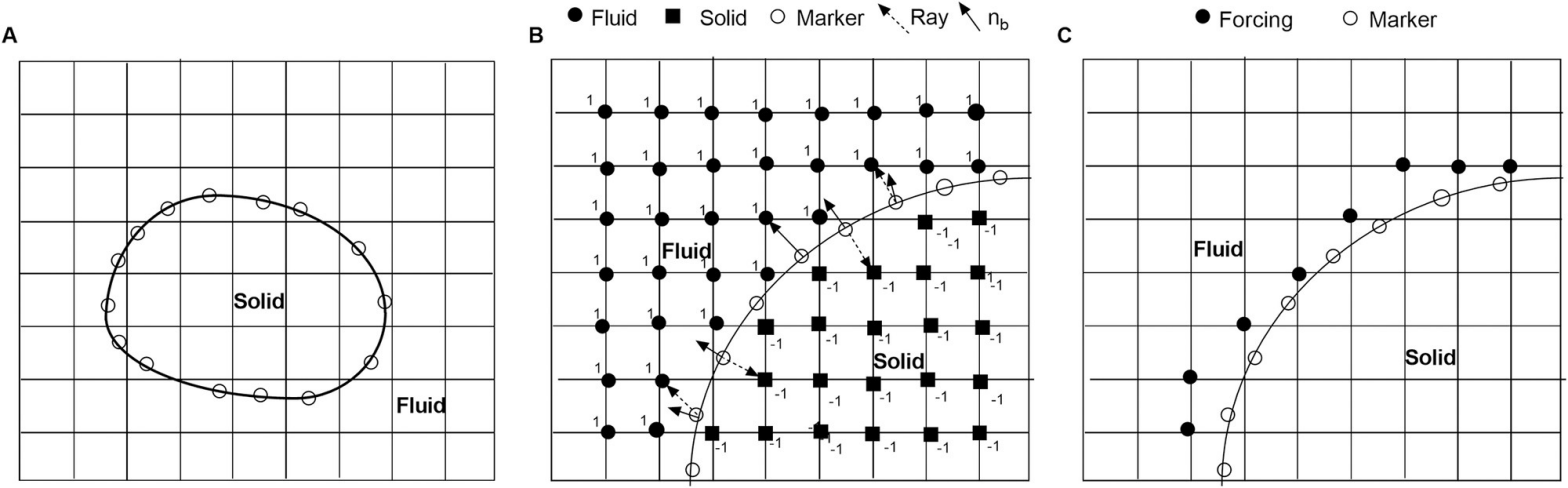
Tracking of solid-fluid interface used in the immersed boundary method. (A) An arbitrary shape solid in an orthogonal computational domain. Open circles indicate marker particles at the boundary which may not coincide with the Eulerian grid points. (B) Identification of solid and fluid points in the computational domain by a ray tracing algorithm. (C) Identification of forcing points (where forcing will be applied) near the boundary after tagging solid and fluid grid points in the computational domain.

A standard second-order central-difference scheme on a staggered grid was used in the present study. In the scheme, the pressure and scalar variables are located at the center of the grid cell, and the velocity components are located at the cell-face center. The solution of Eqs (1) and (2) is obtained by a second-order projection method. Here, time was advanced explicitly using an Adams–Bashforth scheme. An explicit algorithm was used to simplify the implementation of the boundary conditions near the complex boundary. In this two-step time-splitting method, first a provisional value of the velocity field, which is not divergence free, was obtained and used for updating the velocity. The intermediate velocity, ${\hat{u}}_{i}^{n}$ evaluated at the nth step can be written as follows:

$$\begin{array}{c} \frac{{\hat{u}}_{i}^{n}-u_{i}^{n-1}}{\Delta t}=RHS_{i}^{n}+f_{i}^{n} \end{array}$$ (9)

where RHS contains all of the terms on the right hand side of the momentum equation. After solving the Poisson equation, the final velocity, $u_{i}^{n}$, can be updated from the ${\hat{u}}_{i}^{n}$. The external force, $f_{i}^{n}$, needs to be calculated first to implement the desired boundary condition at the boundary point where the boundary may or may not coincide with the grid points. To do this, it is necessary to identify solid, fluid and forcing points near the boundary where forcing functions are implemented in the simulation domain. In the current study, accurate interface tracking methodologies were used for the complex coral geometries. An immersed solid boundary of an arbitrary shape is represented by a series of markers shown in Fig 2. After defining the immersed interface as a series of marker particles, the relationship between these particles and the underlying Eulerian grid can be established by implementing a ray-tracing algorithm. The detailed procedure for identifying solid and fluid points adopted for the current study can be found in [48]. The procedure includes finding the closest grid points near each marker particle. Then, from each marker particle, a ray $\vec{r}$ is shot to the grid point and a normal vector, $\vec{n_{b}}$, is calculated on the marker points. If $\vec{r}\cdot \vec{n_{b}}<0$, then this grid point is inside the solid. Otherwise, it is outside the solid. The forcing points are the closest grid points to the marker points which have at least one solid point near the boundary. A schematic of the procedure for finding solid, fluid and marker points near the boundary is shown in Fig 2.

As mentioned earlier, all of the simulations were performed using STL files of coral geometries, which were each composed of millions of triangles. A ray-tracing algorithm was implemented to establish a relationship between these triangles within an Eulerian grid. The coordinates of the vertices and normal vectors for each triangle are then stored in the STL file. The ray-triangle intersection algorithm used in the reconstruction of the flow field consists of the following steps:

1. A ray is shot from a grid point to a triangle.
2. Determine if the ray intersects the triangle or not.
3. Find the coordinates of the intersecting point.

In the solution procedure, the fluid points are the unknowns and the forcing points are boundary points, while the solid points do not influence the rest of the computation.

If the boundary coincides with the grid points, the forcing function can be calculated directly from the known ${\hat{u}}_{i}^{n}$ at the grid points. Otherwise, the value of velocity at the forcing points can be calculated from the interpolation of surrounding fluid points. The interpolation stencil in this method, however, involves a search procedure for suitable fluid points, and those points in the stencil may contain points that do not belong to the neighboring grid lines of the forcing point. The detailed procedure for identifying the immersed boundary, calculating the forcing function near the boundary, and the interpolation scheme can be found in Balaras *et al*. [48]. Here, the forcing function was calculated only at the boundary.

**Fig 3:**
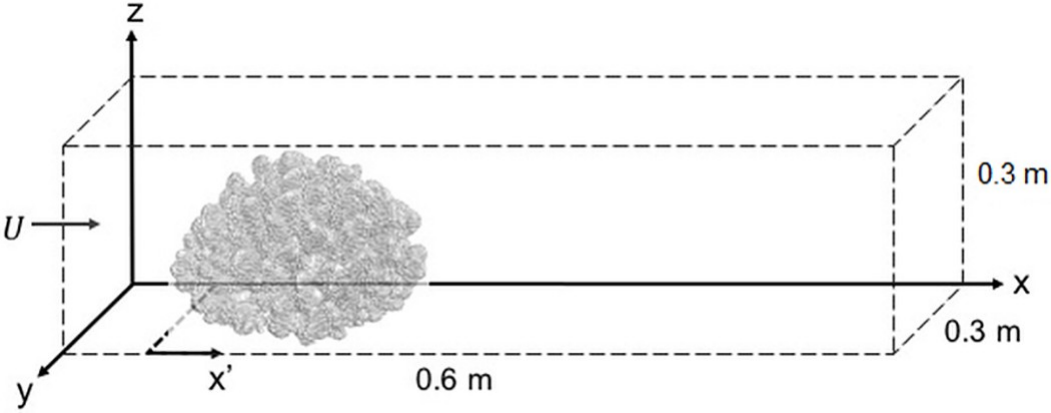
Schematic of the simulation flow domain (not to scale). *x*′ = 0 is the secondary axis used at the beginning of the coral colony. The center of the colony is located at the middle of the flow domain. Uniform grid spacing was used throughout the domain.

The simulation domain for the current study was chosen carefully. Before performing the current analysis, a near-as-possible validation simulation was performed to compare the current results with the experimental results of Chang *et al*. [31] for a high-flow morphology branching coral, *Stylophora pistillata*. For the current analysis, the simulation domain was 0.6 m long in the streamwise direction (*x*), 0.3 m wide in the lateral direction (*y*) and 0.3 m in the vertical direction (*z*), similar to the flow domain used in the experimental and numerical study of Chang *et al*. [31]. As in natural conditions, a single coral sits in the open flow domain with the bottom of the domain representing the ocean floor. But, the flow domain chosen for the simulations to match the experiments of Chang *et al*. is limited in extent and therefore artificially constrains the flow, conditions which will only be approximated in nature for a few rare coral colonies. If the simulation could be performed in an extremely large domain, that would represent the ideal flow scenario of a coral sitting in an open flow environment. At the same time, we have to be realistic regarding the computational expenses when performing a simulation around an extremely complex and arbitrarily shape geometry. In addition, we want to stress the objectives and scale of the current study. We intended to analyze the detailed flow field through and around a single coral colony, where the length scale varies from an individual branch diameter to the length or diameter of the entire colony, and fully three-dimensional LES simulations were used to compute the hydrodynamics in the coral interior at these scales, which are computationally expensive. These constraints have led us to limit the dimensions of the simulation domain. As the current analysis was not intended to resolve diffusion boundary layers less than 1 mm, which would be extremely difficult and computationally expensive for such arbitrary geometries, the current grid resolution of 1 mm in the *x*, *y* and *z* directions was sufficient to meet the objectives of the current study. Similar to Chang *et al*. [37], Dirichlet boundary conditions were applied at the inlet, and an outflow boundary was used at the outlet of the domain. For the lateral direction, slip boundary conditions were implemented at both sides of the coral. Slip boundary conditions were also used at the top face of the domain, and the bottom of the domain was defined as a no-slip wall. In the current analysis, 54 million grid cells were used for all of the simulations. All of the numerical experiments were performed on the Engineering Science and Mechanics (ESM) computing cluster at Virginia Tech. The ESM Linux Computational Cluster (LCC) consists of up to 52 compute nodes with 776 processors, 2TB RAM, and FDR-10 RDMA fabric connecting all of the nodes’ memory. Each of the nodes contains 20 processor cores and 24GB RAM. A schematic of the flow domain is shown in Fig 3.

A few limitations exist in the current study. First, the hydrodynamic analysis preformed on the coral structures was only limited to unidirectional oncoming flow conditions. But oscillatory flows significantly impact the hydrodynamic conditions and biological activities of corals, which live in such flow condition. The literature shows that the nutrient uptake and mass transport rates differ under oscillatory flow conditions [27, 49, 50]. In addition, the branch density comparison was only performed for two *Pocillopora* coral structures. If more coral branching structures with different branching patterns were included in the current study, the detailed study of these structures would provide valuable insights into the impact of branch thickness and inter-branch distance on the flow dynamics at the interior of branching corals. In the current study, it is not possible to resolve the diffusion boundary layer, which is in the order of several hundred microns to 1-2 mm thick at the coral surface [51]. To resolve this thin boundary layer on such complex geometries would require very high spatial resolution on the surface which is extremely expensive and out of the scope of the current study. Finally, the current study would benefit from more rigorous validation with existing experimental results but unfortunately, the availability of detailed three-dimensional flow fields at the interior of corals is limited.

## Results and discussion

**Fig 4:**
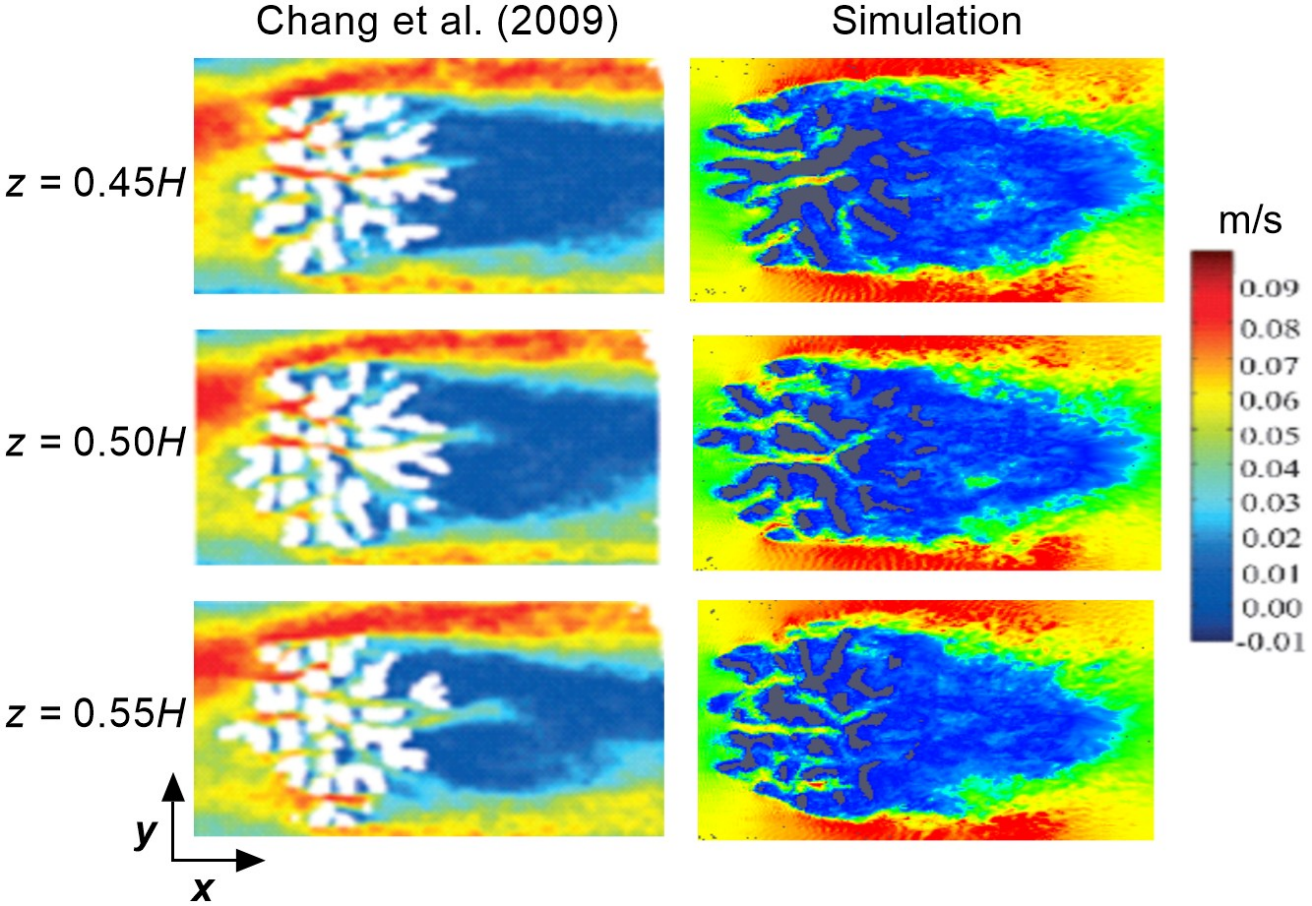
Comparison of top view velocity slices between an experiment and the present simulation at different heights inside the corals. (Left) Top view of velocity slices inside an *S. pistillata* colony obtained by magnetic resonance velocimetry [31] at the colony mid-height (*z* = 0.5*H*, middle slice) and two neighboring slices obtained at 5-mm distances above and below the mid-height level. Reproduced with permission from *Limnology and Oceanography* 54, 1819 (2009). Copyright 2009 by John Wiley and Sons, Inc. (Right) Present LES simulations of the velocity magnitude obtained inside the *P. meandrina* colony at the same normalized height as the experiment.

Before comparing the flow profiles between the two *Pocillopora* structures, the results from the *P. meandrina* simulation were compared with the experimental results of Chang *et al*. [31] for the high-flow morphology of *Stylophora pistillata* for validation. Although every coral in nature is unique, the dimensions of the geometries used in the experiments and the validation simulation were extremely similar. In the experiment, the streamwise (*x*), cross-stream (*y*), and vertical (*z*) extents of the densely-branched coral were 0.128 m, 0.132 m and 0.09 m, respectively. In the simulation, the extents of the densely-branched coral were 0.172 m, 0.172 m and 0.1 m, respectively, in the stream-wise, lateral and vertical directions. The detailed physical and morphological properties of the two coral geometries are shown in Table 1. The channel dimensions for the experiment and validation simulation domains were also similar. In the experiment, the bulk velocity in the channel was 0.05 m/s and the Reynolds number, based on the diameter of the branch, was 580. Based on the height of the coral, it was approximately 5, 000. A similar Reynolds number was used in the simulation to keep the flow conditions close to the original experiment. A qualitative comparison of the experimental and the simulation result is shown in Fig 4. The figure compares the cross-sectional velocity profiles (top view) obtained in both corals at the different heights inside the corals. For the experiment, the middle slice was obtained exactly at the mid-height of the coral and the other slices were obtained at a 5-mm distance in height from the center slice. In the simulation, the slices were obtained at the same locations. Relatively high velocity magnitudes were observed around coral exterior in both cases, and distinct wake regions formed behind both colonies. Because of the dense branch structure, the colony largely acted as a baffle and most of the flow was diverted outward and vertically upward. The dense branching significantly reduced the velocity in the rear section. Although the oncoming velocity profile in Chang *et al*. is somewhat skewed owing to a curved, recirculating water channel, both velocity fields demonstrate the same basic characteristics for these flow conditions. The results also demonstrated a low velocity region behind the thick branches of the coral and the velocity magnitude was almost identical for both cases.

**Table 1:** Physical properties of the coral colony used in Chang *et al*. and in the current simulation.

Colony information	Chang *et al*.	Current simulation
Coral species	*S. pistillata*	*P. meandrina*
Ratio of volume to surface area	2.0	2.0
Ratio of frontal to surface area	0.078	0.0754
Ratio of branch distance to diameter	4.5	3.0 to 4.0

**Fig 5:**
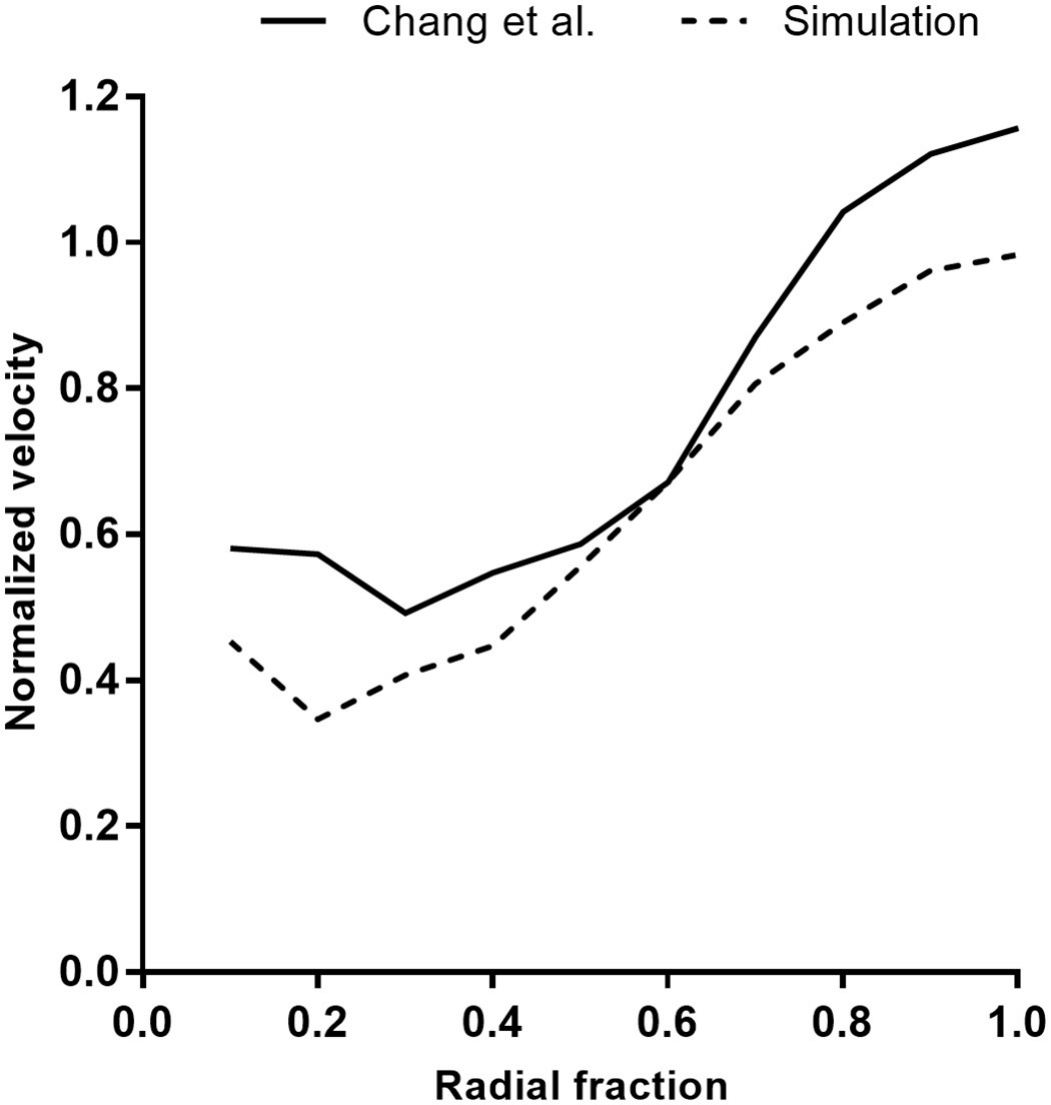
Comparison of normalized mean shell velocity between an experiment and the present simulation. The mean shell velocity is plotted as a function of radial distance from the center of the colony to the exterior and normalized by the oncoming velocity magnitude. The difference in the mean shell velocity profiles may be due to the difference in the structures’ branch distance to branch diameter ratios, which are reported in Table 1. The experimental data is obtained by magnetic resonance velocimetry in a *S. pistillata* colony [31]. Reproduced with permission from *Limnology and Oceanography* 54, 1819 (2009). Copyright 2009 by John Wiley and Sons, Inc.

A quantitative comparison was also performed to determine the variation in the average velocity between the experimental results of Chang *et al*. [31] for the high-flow morphology of *Stylophora pistillata* and the current numerical simulations, in order to further validate the simulations. In the experimental analysis, the variation of the velocity at the interior of the colony was plotted as a function of radial distance from the center of the coral. We used the same approach for the computational analysis in order to compare the results. The center of the coral was selected by the midpoint of the *x*, *y* and *z* extents of the colony and the mean velocity was obtained at different radial distance from this point. Fig 5 compares the mean velocity magnitude from the interior to the exterior of the colony in both studies. In general, the two profiles show similar velocity trends from exterior to interior. Except a the mid-radius of the colony, the computational profile shows lower velocity magnitudes than the experimental analysis though the overall trend remains same. One reason for this discrepancy might be the difference of the compactness of the branch density between these two geometries. In terms of the ratio of inter-branch distance to branch diameter, the geometry used in the computational study has a lower ratio than the experiential geometry, which results in higher flow resistance inside the colony.

**Fig 6:**
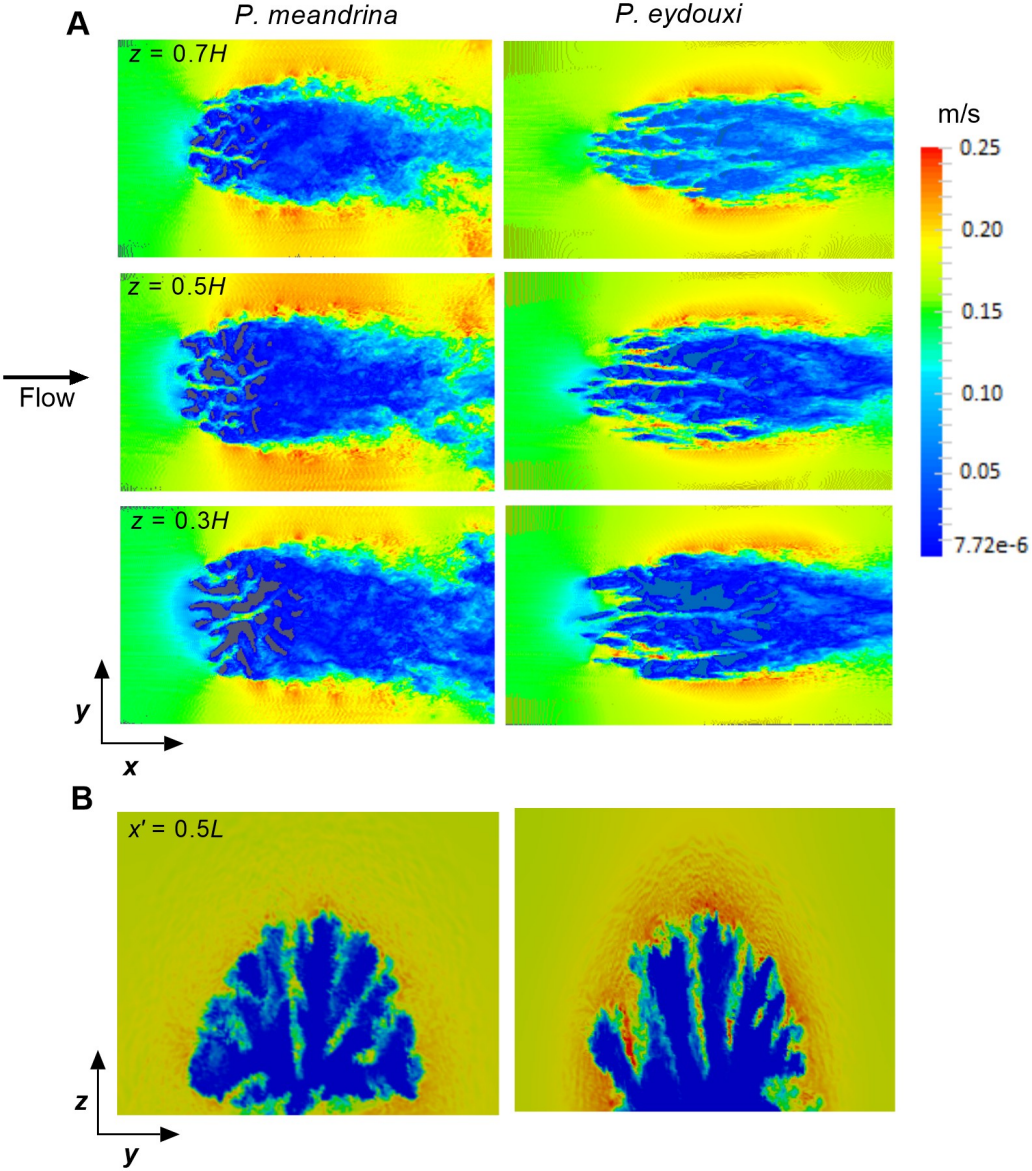
Comparison of top view velocity slices between two *Pocillopora* colonies with different branching structures. (A) Top view of velocity slices inside *P. meandrina* (left) and *P. eydouxi* (right) at *z* = 0.3*H*, 0.5*H*, and 0.7*H* above the base of the coral for the same oncoming velocity of 0.15 m/s, where *H* is the height of the colony. (B) Comparison of front view velocity slices between *P. meandrina* (left) and *P. eydouxi* (right). These slices were obtained at the middle of the coral (*x*′ = 0.5*L*) along the flow direction, where *L* is the length of the colony.

The flow profile inside the coral is a function of the inter-branch distance, branch diameter, coral height, and oncoming velocity condition. To understand the effects of branch density on the surrounding flow, the flows through two different coral species with distinct branching patterns from the same *Pocillopora* coral genus were simulated and compared for the same oncoming flow condition. The simulation parameters for these the two coral geometries are shown in Table 2. Top views of flow through these two *P. meadrina* corals are shown in Fig 6 for an oncoming uniform flow of magnitude 0.15 m/s. The oncoming velocity was selected based on the flow conditions used for the numerical experiments perfromed by Chang *et al*. on *Pocillopora* coral [37]. The middle velocity slice was obtained at approximately the midsection of the coral and the rest of the slices were obtained at 20-mm intervals on both sides of the middle slice. For both corals, a stagnation region formed near the front section, and the effect was higher for *P. meandrina* due to its higher branch density. For *P. meandrina*, higher velocity values were observed at the top and both sides of the colony and the velocity reduced substantially and became almost uniform in the latter half of the coral. Most of the flow is diverted to the top and sides of the colony for *P. meandrina*, demonstrating the relatively higher flow restriction in this colony due to its compactly spaced interior branches. Mixing and transport take place at the coral surface, mediated by the flow. At the downstream end of the *P. meandrina* colony, a distinct wake region containing a large recirculation zone formed behind the colony. In contrast, there was higher flow penetration at the interior of *P. eydouxi* compared to *P. meandrina*. The more open colony geometry of *P. eydouxi* contained higher flow rates in the interior. Though a large portion of the flow was still diverted outward, the penetration was much better in *P. eydouxi* than *P. meandrina*.

**Table 2:** Simulation parameters for *Pocillopora*. Here Reynolds number was calculated based on coral height.

Coral species	Flow	*U*_*incoming*_(*m*/*s*)	Re
*P. meandrina*	Unidirectional	0.15	15,000
*P. eydouxi*	Unidirectional	0.15	15,000

For qualitative measurements of the variation of velocity profiles along the lateral direction, a velocity slice (perpendicular to flow) was taken at *x*′ = 0.5*L* for both corals. Fig 6B shows a comparison of velocity slices obtained at the middle of the coral, perpendicular to the flow direction. For both of the corals, the outer periphery shows higher velocity magnitudes due to the constraints of the flow domain. For the same oncoming flow conditions, *P. meandrina* shows relatively low velocity magnitude at the coral interior and the oncoming flow loses most of its momentum due to high branch density. In comparison, *P. eydouxi* shows relatively higher velocity magnitudes between the branches. These higher velocity magnitude can be observed mainly at the narrow corners between the branches. As the area at these corners reduced, the velocity magnitude increased due to the conservation of mass.

**Fig 7:**
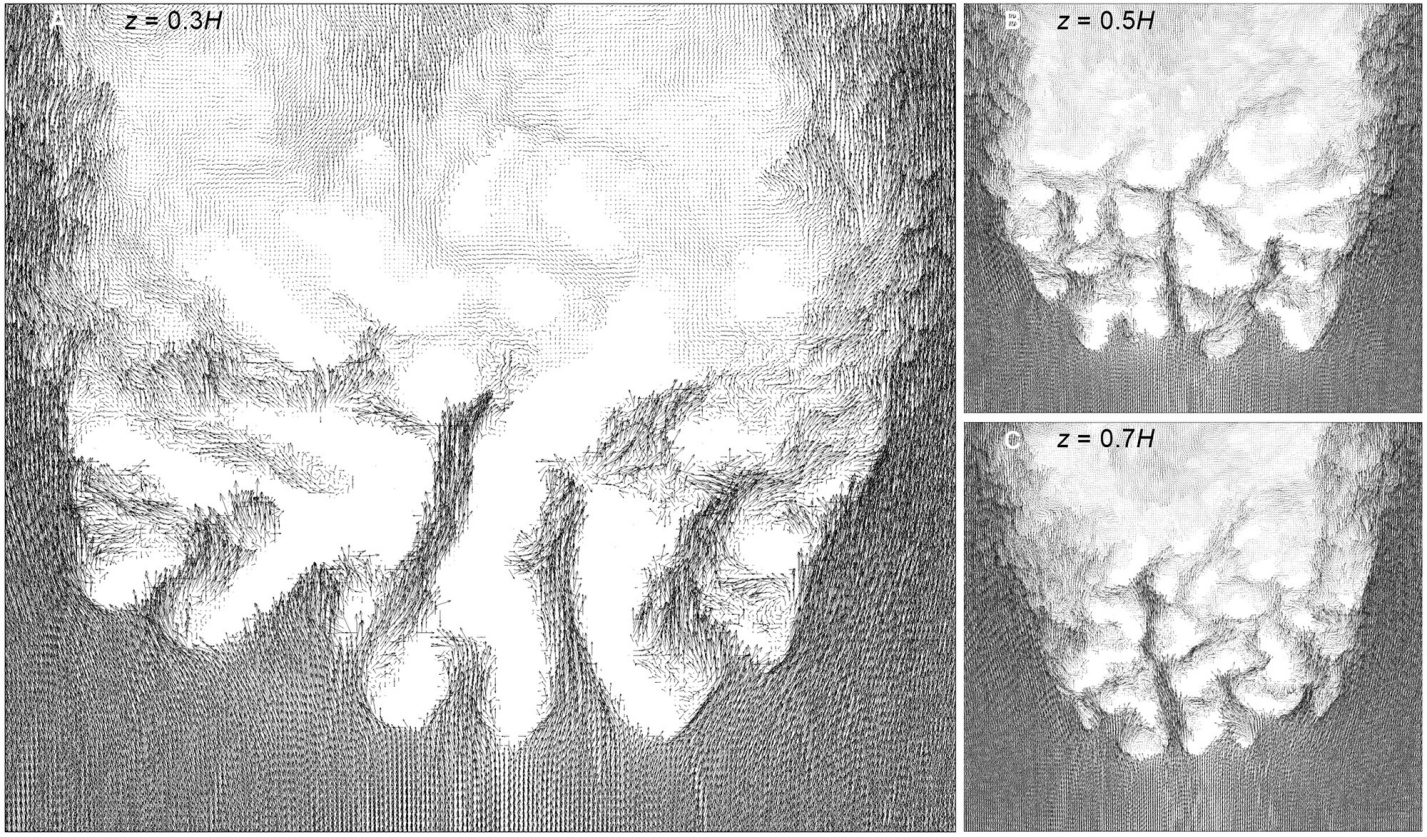
Top view of velocity vector fields at three heights inside the *P. meandrina* colony. The vector fields were obtained at heights *z* = 0.3*H* (left), 0.5*H* (top right), and 0.7*H* (bottom right) above the base of the coral colony, where *H* is the colony height. The figures show higher velocity magnitudes at the anterior end of the colony and allow one to gauge how far the oncoming flow can penetrate easily into the colony before it is slowed substantially by frictive contact with the coral branches.

**Fig 8:**
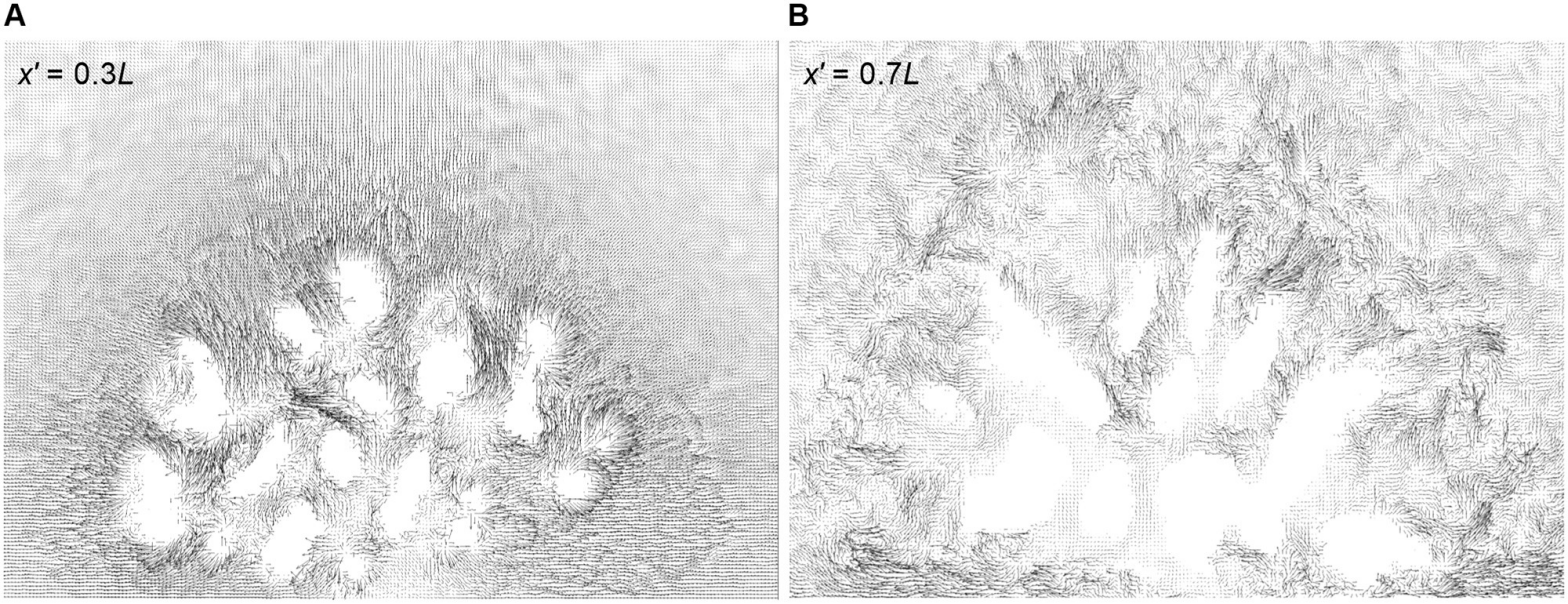
Front view of velocity vector fields at anterior and posterior cross sections of the *P. meandrina* colony. The vector fields were obtained at an upstream position inside the coral colony (*x*′ = 0.3*L*, left) and at a downstream position (*x*′ = 0.7*L*, right) relative to the front edge of the colony, where *L* is the colony length in the streamwise direction. The downstream vector field shows the formation of large vortices at the periphery of coral.

**Fig 9:**
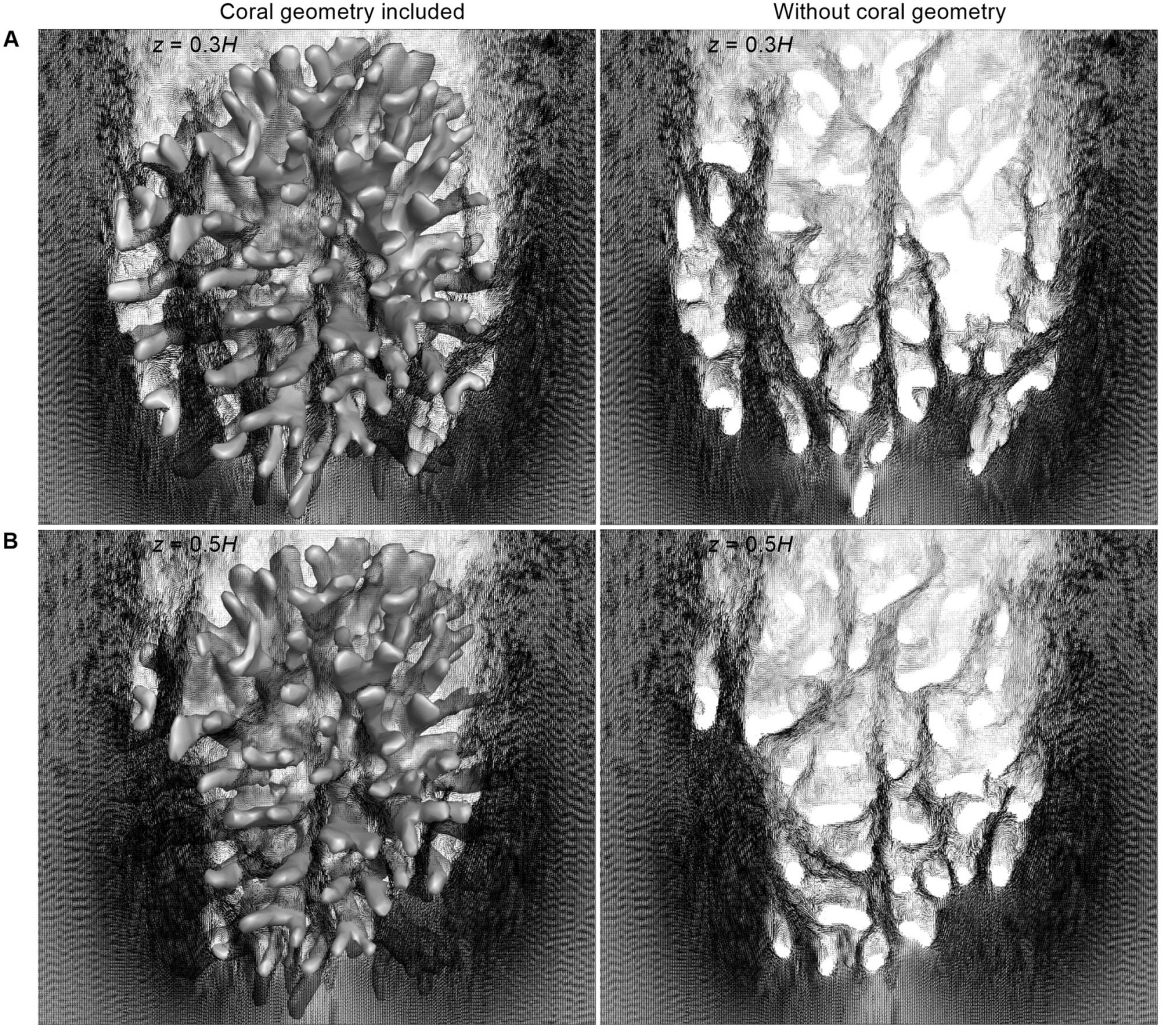
Top view of velocity vector field at two different heights inside the *P. eydouxi* colony, with and without the coral geometry. (A) The vector field obtained at a height of *z* = 0.3*H* with the coral colony geometry included in the image (left) and without the geometry (right). (B) The vector field obtained at a height of *z* = 0.5*H* with the coral colony geometry included in the image (left) and without the geometry (right). The figures show the formation of wakes behind the individual branches in the colony, and better flow penetration can be seen at the interior of colony compared to *P. meandrina*.

Vector profiles at the interior of colony are shown in Fig 7. The top view of velocity vector fields inside *P. meadrina* is shown at different height above the base of the colony. From the vector profiles at *z* = 0.3 & 0.5*H* it can be clearly observed that the anterior part of the colony has higher velocity magnitudes than the posterior half. Two streamwise velocity vector field sections from the front and rear sections of the *P. meadrina* colony are shown in (Fig 8). We note the formation of vortices at the outer periphery of the colony. These external vortices stir the water column and enhance the mass transport at the exterior of colony. Horizontal velocity vectors fields are shown at different heights from the base of the colony in (Fig 9). These vector fields show substantial differences between the two structures. Because of the more open branch spacing, the formation of wake zones can be observed behind each branch in *P.eydouxi*. The formation of these recirculating zones may help *P.eydouxi* by keeping the traveling solute in these zones for longer times, which enhances the transport process.

**Fig 10:**
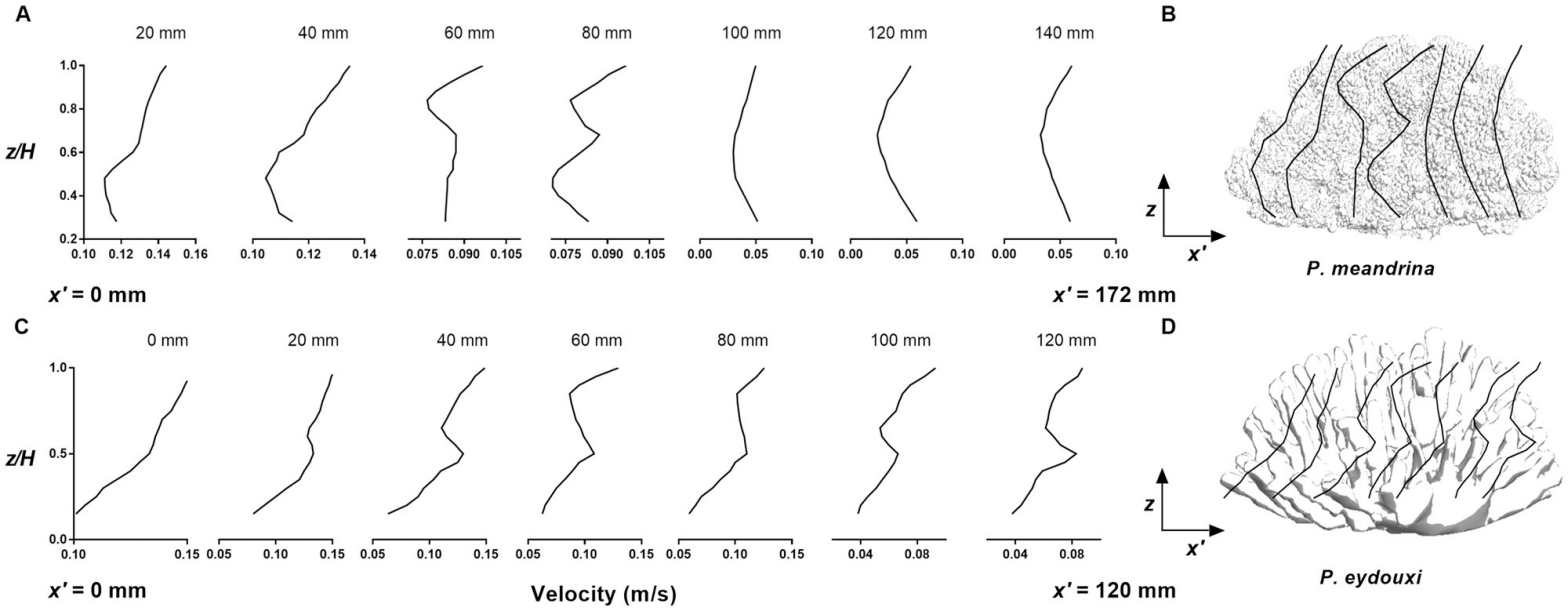
Streamwise variation of the mean streamwise velocity profile in *P. meandrina*. (A and C) Mean streamwise velocity profiles in the *P. meandrina* and *P. eydouxi* colonies, respectively, as a function of normalized height at 20-mm intervals along the length of the colony. The profiles were obtained by averaging the velocity magnitudes of the closest 10 neighboring grid points at each location in the *x*-*z* planes, and then averaging the velocity magnitudes over the lateral (*y*) direction within the colonies. (B and D) Schematic of the locations of the mean velocity profiles within the coral colonies (not to scale).

Up to now, we have provided qualitative comparisons of flow structure between two *Pocillopora* colonies. But for better comparison, we require quantitative measurements of differences in the mean flow characteristics between these two corals. To attain this objective, mean velocity profiles were obtained as a function of coral height inside both of the corals at 20-mm intervals. As the branches are situated randomly, it is difficult to obtain mean velocity profiles at different sections inside the coral. To overcome this issue, these velocity profiles were calculated by averaging the velocity magnitudes of the closest 10 neighbor grid points at each location in the *X* − *Z* planes, and then a second averaging procedure was performed in the lateral (*y*) direction within the coral. These velocity profiles not only depicted the progression of flow inside the coral, but also indicated the amount of mixing and transport that takes place along the length of the coral. The mean velocity profiles obtained for both *Pocillopora* corals are shown in Fig 10A and 10C. For *P. meandrina*, the mean velocity profiles were similar in character up to 40 mm into the colony. This is the anterior portion of the colony, where the flow penetration is high. The maximum velocity dropped to approximately 33% of oncoming velocity in this region. For 60 to 80 mm, the flow decelerated and dropped to a maximum 50% of the oncoming flow magnitude. In this middle region, the velocity profiles changed substantially. Interestingly, at 80 mm, the profile displayed a velocity peak at the coral mid height, indicating a jetting flow structure. From 100 mm until the end of coral, the velocity profiles were almost identical and displayed a reduction in velocity magnitude at mid-height of the coral. In general, the velocity profiles showed low velocity at the mid height of the coral and comparatively higher velocity at the top and bottom sections of the coral.

In contrast, the velocity profiles within *P. eydouxi* remained similar in character from front to the back of the colony, even though the velocity dropped substantially at the rear of coral, due to the relative openness of the structure. The magnitude of the flow within the top half of the coral was comparatively higher than in the lower half of the coral. Around *z* = 0.5*H*, the mean velocity profile shows a small jetting region due to the accelerated flow in the reduced area between the branches at lower section of the coral. At the rear section of coral, between 80 and 120 mm, the maximum velocity dropped to almost 50% of the oncoming flow value.

**Fig 11:**
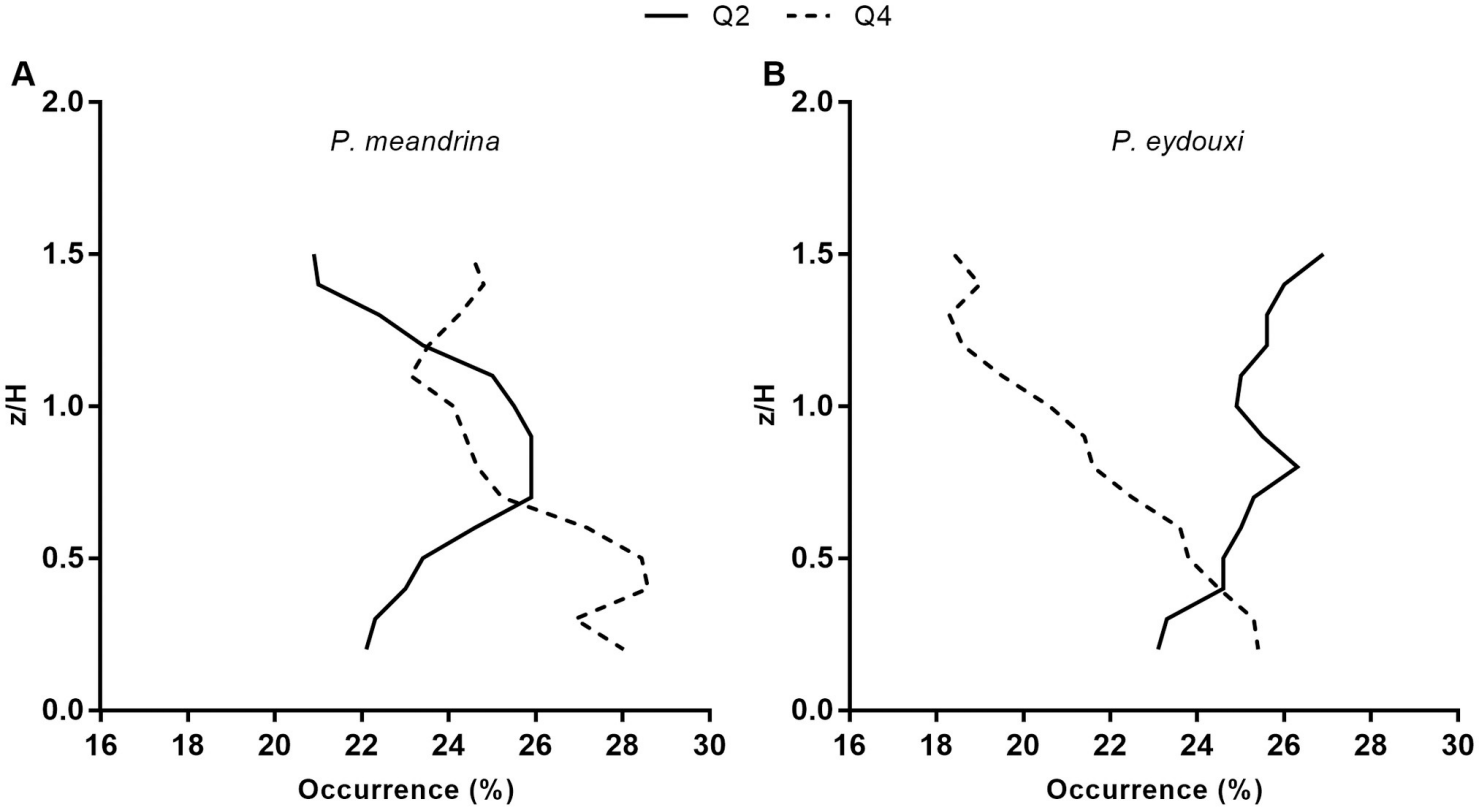
Quadrant analysis performed as a function of coral height for both *Pocillopora* colonies. (A) Ejection (Q2) and sweep (Q4) events and their relative contributions to the Reynolds stress for the *P. meandrina* colony. (B) Ejection (Q2) and sweep (Q4) events and their relative contributions to the Reynolds stress for the *P. eydouxi* colony. The Q2 and Q4 values in the *P. eydouxi* colony diverge at the top of the colony and above it, while those for the *P. meandrina* colony do not.

Finally, we computed the fluctuating parts of all the velocity components and performed a standard quadrant analysis to quantify the interactions of ejections (Q2) and sweep events (Q4) and their relative contributions to the Reynolds stress at the interior of colony. Here streamwise and vertical velocity fluctuation were grouped into quadrants. Fig 11 compares Q2 (*u*′ < 0 & *w*′ > 0) and Q4 (*u*′ > 0 & *w*′ < 0) as a function of coral height for both coral structures. For densely branched *P.meandrina*, the dominance of Q2 over Q4 was observed at the exterior of colony and mixed zones were observed at the interior of colony. Similar profiles for Q2 and Q4 have been reported by Asher *et al*. for the same *P. meandrina* colony geometry in a flume study performed on multiple coral colonies [52]. In contrast, the contributions of Q2 and Q4 to the Reynolds stress is quite different in *P.eydouxi* within the branches of colony. Here, the ejection contributes more than the sweep above a height of *z* = 0.4*H*.

### Flow dynamics over *M. capitata* with and without roughness elements

**Fig 12:**
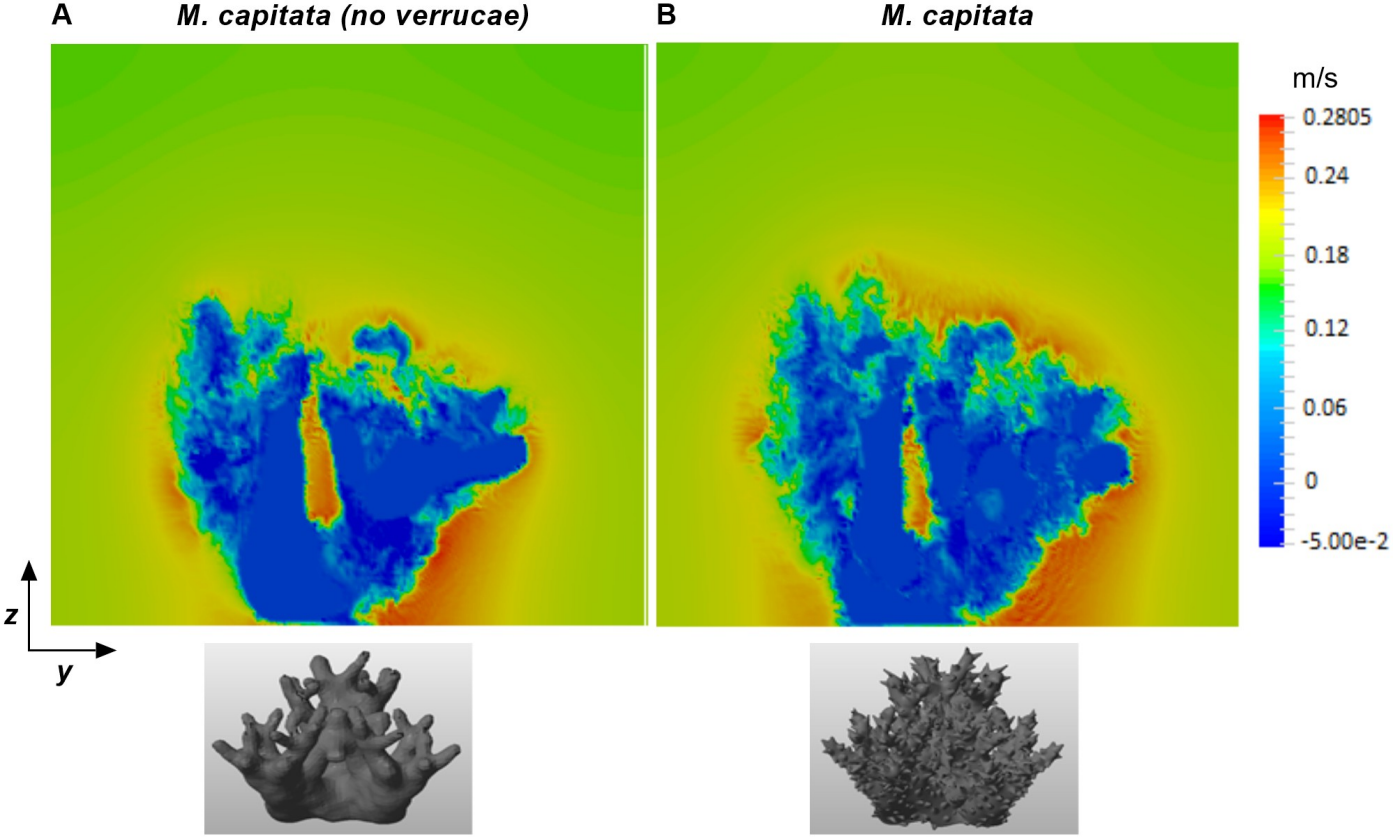
Front view velocity slices in *M. capitata* colonies with and without verrucae. (A) A front view velocity slice at *x*′ = 0.7*L*, where *L* is the colony length in the streamwise direction, for the *M. capitata* colony with the verrucae removed. The verrucae were removed from the colony surface by smoothing the STL geometry file while keeping the other geometric features the same. (B) A streamwise velocity slice at *x*′ = 0.7*L* for the *M. capitata* colony with the verrucae intact. Both simulations were performed at a Reynolds number of 15, 000.

For corals, the nutrient transfer to the colony and the transport of dissolved gases away from the coral surface depend on the shear profile, turbulent mixing, and the concentration gradient near the surface. Quantifying the turbulent stresses is essential for explaining mixing, and stress and drag developed on the coral surface. Even small changes in flow conditions can change these turbulent statistics over the boundary layer. As discussed in the Introduction, *M. capitata* with verrucae appearing on its surface grows near Kanehoe Bay in Hawaii. To ascertain the impact of verrucae in naturalistic flow conditions, a computational analysis was performed on two instances of the same loosely-branched coral *M. capitata* colony, one with the naturally occurring verrucae intact, and one with the verrucae removed. The colony geometries used can be seen at the bottom of (Fig 12). To characterize the boundary layers and the turbulent stresses that developed on these structures, velocity profiles were computed over the coral surface at two different Reynolds numbers, 5, 000 and 15, 000, which were based on the low and high water velocity values near Kanehoe Bay (velocity magnitude was selected based on one week flow information near the area at -0.25m depth) [53] and the height of the coral. For ease of discussion, we have used the abbreviations “CV” and “CWOV” for the corals with and without verrucae, respectively. Fig 12 shows slices of the flow field around *M. capitata* with and without verrucae at Reynolds number 15, 000. After computing flow fields for both structures, the velocity and stresses were calculated above the surface of the corals, at the location at *x*′ = 0.7*L*, at two different Reynolds numbers. Then, the velocity and stress profiles obtained were averaged along the lateral (*y*) direction within coral to obtain mean profiles at the coral’s top surface. The mean profiles were compared for the CV and CWOV cases for the same flow conditions, in order to investigate the effects of the verrucae on the flow field.

**Fig 13:**
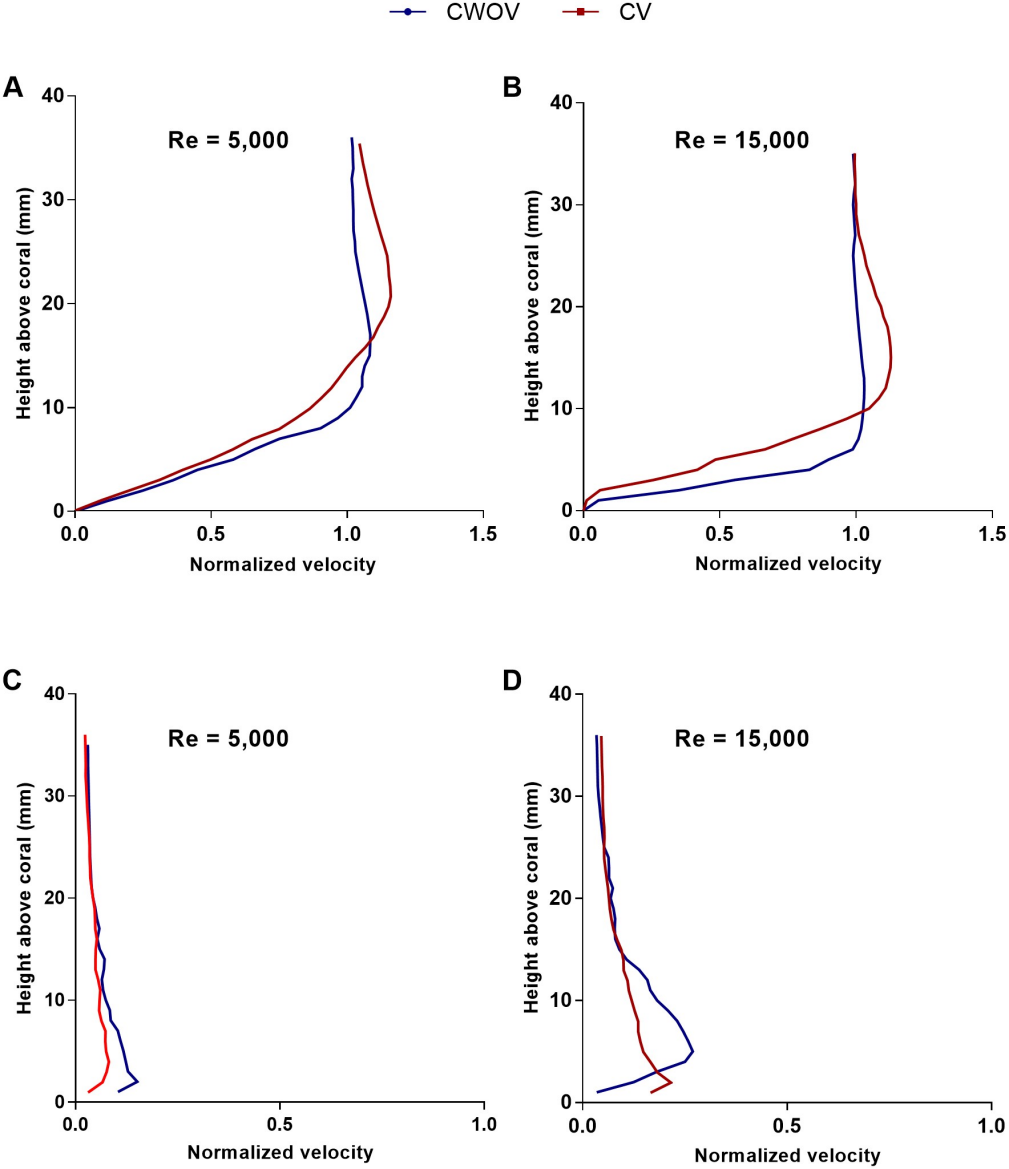
Velocity profiles above the *M. capitata* colony with and without verrucae. (A) Normalized streamwise velocity profiles for the CV and CWOV colonies at Reynolds number 5, 000. (B) Normalized streamwise velocity profiles for the CV and CWOV colonies at Reynolds number 15, 000. (C) Normalized vertical velocity profiles for the CV and CWOV colonies at Reynolds number 5, 000. (D) Normalized vertical velocity profiles for the CV and CWOV colonies at Reynolds number 15, 000. The velocity profiles were obtained at the top surface of the coral at *x*′ = 0.7*L*, where *L* is the colony length in the streamwise direction, and averaged are over the lateral (*y*) direction. The Reynolds number was calculated based on the height of the colony. The velocity profiles were normalized by the oncoming flow speed.

Fig 13A and 13B compare the streamwise velocity profiles at the top of *M. capitata* with and without verrucae at Reynolds numbers 5, 000 and 15, 000, respectively. Here, the mean velocity profile was normalized by the oncoming velocity magnitude. At both Reynolds numbers, very similar flow profiles exits above the colonies. The maximum velocity for CV, though, was located at almost twice the height above the colony surface than than for CWOV. In contrast, there were significant differences in the streamwise velocity profiles between the two geometries at Reynolds number 15, 000. At this Reynolds number, the streamwise velocity reached 50% of its maximum magnitude at about 2 and 5 mm above the surface of the CWOV and CV corals, respectively. Recently, Reidenbach and Stocking *et al*. [43, 54] measured streamwise velocity components at the top of a single coral colony under unidirectional flow conditions and their results show similar velocity profiles at the top of the colony, with similarly located profile inflection points.

Corals depend on the surrounding flow for the transfer of nutrients, removal of waste, and mass transport. High velocity magnitudes near the coral surface indicate fast mass and momentum transfer to and from the coral surface [27, 49]. This is very important for biological processes like photosynthesis and respiration which depend on the thickness of the diffusion boundary layer (DBL) on the surface of coral. Experimental studies show that the thickness of the DBL decreases with an increase in flow magnitude [51]. If a comparison is made between the CWOV and CV colonies, the mixing and transport should be better for the CWOV colony at both Reynolds numbers, due to the higher streamwise velocity magnitudes near the coral surface.

Fig 13C and 13D compare the vertical velocity profiles at the top of *M. capitata* with (CV) and without verrucae (CWOV) at Reynolds numbers 5, 000 and 15, 000, respectively. At both Reynolds number, the vertical velocity magnitudes were relatively small in comparison to the streamwise velocity components. At Reynolds number 5, 000, the vertical velocity profiles were similar for both colonies. At the larger Reynolds number, the velocity profile demonstrated a relatively higher magnitude near the top of the CWOV colony. In both cases, the vertical velocity profiles reached a constant magnitude at approximately 15 mm above the coral surface. If we compare the streamwise velocity profiles to the vertical profiles, the mass and momentum transport is dominant in the horizontal direction at both Reynolds numbers. If the vertical flow profiles for these two coral structures are compared, the CWOV colony should have better mixing and transport along the vertical direction than the CV colony. Also, the nutrient gradient above the canopy will be more uniform for the CWOV colony due to the comparatively higher vertical flow rate.

#### Turbulent stresses at the coral surface

**Fig 14:**
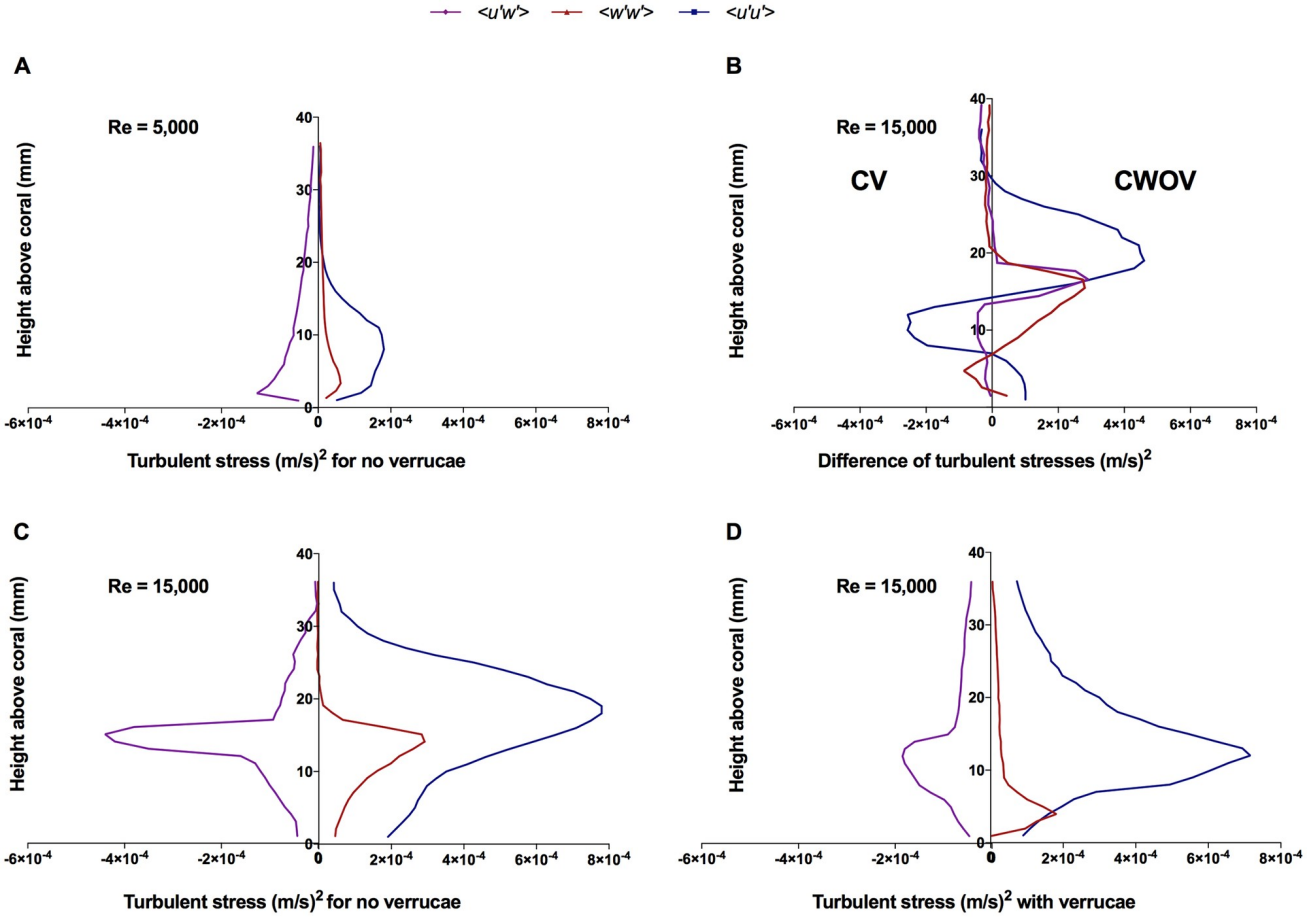
Turbulent stress above the *M. capitata* colony with and without verrucae. (A) The turbulent stresses above the *M. capitata* colony without verrucae at Reynolds number 5, 000. (B) The difference between the turbulent stresses above *M. capitata* with and without verrucae at Reynolds number 15, 000. (Subfigure C minus Subfigure D.) (C) The turbulent stresses above the *M. capitata* colony without verrucae at Reynolds number 15, 000. (D) The turbulent stresses above the *M. capitata* colony with verrucae at Reynolds number 15, 000. All of the profiles were obtained above the top surface of the coral at a streamwise position of *x*′ = 0.7*L* and were averaged over the lateral (*y*) direction.

The transfer of nutrients and mass between corals and their overlying water column also depends upon the turbulent stresses developed at the coral surface. The Reynolds stress, 〈*u*′*w*′〉, is mainly responsible for mixing near the coral surface. To calculate these values, time-and-space averaged turbulent stress components 〈*u*′*u*′〉, 〈*w*′*w*′〉, and 〈*u*′*w*′〉, were computed at the top of the *M. capitata* colony with and without verrucae for unidirectional oncoming flow conditions at two different Reynolds numbers. To capture the fluctuating quantities, mean values were subtracted from instantaneous values of the velocity components and the simulation was given enough time for the mean flow to stabilize. Fig 14A shows turbulent stresses developed at the top of *M. capitata* without verrucae (CWOV) at Reynolds numbers 5, 000 and Fig 14C & 14D show the turbulent stress over *M. capitata* with and without verrucae at Reynolds 15, 000, respectively. The difference of the magnitude of these stress components over the same pair of geometries were plotted as a function of height in Fig 14B at Reynolds number 15, 000.

At Reynolds number 5, 000, the 〈*u*′*u*′〉 turbulent stresses showed higher magnitudes than 〈*w*′*w*′〉 and 〈*u*′*w*′〉 and reached maximum values at approximately 9 mm above the coral surface, indicating the location of the maximum streamwise momentum near the coral polyps on the coral surface. All the turbulent stress components approached zero magnitude at a height of approximately 20 mm above the coral surface and the maximum magnitude of Reynolds stress was approximately 50% of the maximum value of the 〈*u*′*u*′〉 stress. In contrast, at the higher Reynolds number of 15, 000, the maximum magnitude of 〈*u*′*u*′〉 stress increased to almost four times that for Re 5, 000, with the maximum value of all the stress components occurring at approximately 15 mm above the top of the coral. At the higher Reynolds number, the maximum magnitude of Reynolds stress, 〈*u*′*w*′〉, increased significantly, which would contribute to elevated mixing and vertical transport due to high shear at the top of the coral.

Fig 14D shows the components of the turbulent stresses developed on top of *M. capitata* with verrucae (CV) at Reynolds number 15, 000. Here, the value and location of the maximum magnitude of the 〈*u*′*u*′〉 stress was similar to streamwise stress component developed on CWOV. For the vertical stress component, 〈*w*′*w*′〉, CWOV shows greater magnitude than CV.

Similarly for the Reynolds stress, the maximum magnitude of 〈*u*′*w*′〉 for the CWOV colony was almost twice that of the CV colony. To provide validation support for these results, these turbulent profiles were compared with the turbulent stresses measured on top of a single coral colony from the experimental study by Stocking *et al*. [54]. In the experimental study, the maximum magnitude of the turbulent stresses takes place within a 20 mm height above the top surface of the coral. In the case of our simulation, the maximum turbulent stress components were also found within a 20 mm height above the coral surface, and the magnitude of the stress components are within the same order of magnitude as those found in the experiment. In the experiment, the vertical stress, 〈*w*′*w*′〉, was almost half of the streamwise turbulent stress, 〈*u*′*u*′〉, but in the simulation, comparatively lower vertical stress was observed except for in the CWOV colony at Reynolds number 15, 000. For a better understanding of the impact of verrucae on turbulent stresses, the difference of their magnitude were plotted as a function of height above the top surface of *M. capitata* at Reynolds number 15, 000 in Fig 14B.

In general, the maximum magnitude of the Reynolds stress can be found just at the top surface the of colony for both the CV and CWOV structures, and the value then decreases with the height. Similarly, the maximum magnitudes for 〈*u*′*u*′〉 and 〈*w*′*w*′〉 were located close to the top of both structures. We were also able to capture some distinct features of the turbulent stresses which have direct implications for the biological activities of the corals. For example, calculating the bed shear stress, which can be computed as *t*_*b*_ = −*ρ*〈*u*′*w*′〉 ([55]), indicates that the magnitude of the bed stress will be higher at the top of the coral for the CWOV structure than for the CV structure under unidirectional flow conditions because of relatively higher Reynolds stress. The Reynolds stress can be also used to calculate the friction velocity above the coral. Friction velocity is an important quantity for characterizing the stresses in the overlying water column above the coral. In canopy flow analysis, the friction velocity is calculated as $u_{*}=\sqrt{\langle u^{\prime }w^{\prime }\rangle }$ where the Reynolds stress used is the maximum magnitude within the canopy [25]. Comparing the Reynolds stress profiles for both colonies, it can be concluded that the CWOV structure has a higher friction velocity above the colony than the CV structure. The drag coefficient also depends on the friction velocity, u_*_ and is defined by $C_{d}=\frac{u_{*}^{2}}{U_{0}^{2}}$ where *U*_0_ is the reference velocity [42]. Since both analyses were preformed on *M. capitata* for the same oncoming flow condition, the drag coefficient will be higher for the CWOV structure since u_*_ is higher above this structure. Similarly, the local mass flux per unit area from the fluid to the sediment-water interface can be expressed as *m* = *k*(*C*_*f*_ − *C*_*s*_) where *K* is the local mean mass transport coefficient [56]. The linear relationship between *K* and u_*_ suggests that for the same concentration gradient between the coral surface and the surrounding fluid, the CWOV structure will have a higher mass transfer rate than the CV structure.

## Conclusions

Computational studies were performed in order to understand the differences in mean flow profile characteristics inside two *Pocillopora* coral colony geometries with different branching patterns, one comparatively loosely branched (*P. eydouxi*), and one densely branched (*P. meandrina*). Velocity magnitude and vector fields were presented at different cross section of the colonies to depict flow around the coral branches. Horizontal velocity slices showed higher velocity magnitudes at the anterior end of both colonies compared to the posterior end. To quantify the differences in mean flow characteristics between the two colony geometries, mean velocity profiles were plotted as a function of coral height from the front to back of the colonies. For the dense geometry, *P. meandrina*, the mean velocity profile shows distinct changes through the interior of coral. The anterior part of the colony, which faces the oncoming flow, shows higher velocity magnitudes at the top and base of the colony, with a velocity deficit at mid-colony heights. The dense branching pattern presents higher resistance to flow in the middle part of the colony, resulting in a peak in the mean velocity profile at the colony mid height which may represent jetting. Finally, the mean velocity profile loses the mid height peak in the posterior third of the colony, and the profile recovers the overall shape it had in the anterior third of the colony. The mean normalized velocity along the length of the coral drops to approximately 38% of the freestream value in the middle of the coral, but recovers to 65% at the rear of the coral. In contrast, the mean velocity profiles remain largely uniform throughout the loosely branched geometry, *P. eydouxi*. These profiles have their maximum magnitude at the top of the colony and decrease in value approximately linearly with a decrease in height. Additionally, quadrant analyses was preformed on both these structures to understand the impact of injection and sweep on the Reynolds stresses at the interior of colony.

Simulations were also performed on a *M. capitata* colony geometry with and without verrucae at two different Reynolds numbers to understand the impact of verrucae on the local hydrodynamics. The results displayed distinct inflection points and differences in the mean streamwise velocity profiles for the colonies with and without verrucae. At both Reynolds numbers, the maximum magnitude of the mean streamwise velocity can be observed closer to the top surface of the CWOV colony than the CV colony. Similarly, the mean vertical velocity shows a relatively higher magnitude nearer to the top of the CWOV structure. Higher velocity magnitudes near the surface of the colony ensure better mass transport, so we expect to see higher rates of mass transport from the surface of the colony without verrucae. In addition to velocity profiles, the turbulent stresses were also calculated for both colony morphologies. The profiles of all the turbulent stress components displayed maximum magnitudes just above the top surface of the colonies. For both structures, the streamwise stress, 〈*u*′*u*′〉 showed similar magnitudes for the same oncoming flow conditions. But significant differences were found between the Reynolds stresses, 〈*u*′*w*′〉, developed on the top surface of the colonies. For the CWOV structure, the maximum magnitude of 〈*u*′*w*′〉 was almost twice than for the CV structure. The difference in the turbulent stresses was plotted at the top of *M. capitata* with and without verrucae. This showed higher turbulent stresses for the CWOV structure than the CV structure. From the comparisons of the mean velocity profiles and turbulent stresses it can be concluded that the CWOV structure will experience higher bed stress and friction velocities above the colony than will the CV structure. The higher friction velocity values for the structure without verrucae means it will have a larger drag coefficient and better mass transport for the same oncoming flow condition compared to the structure with verrucae. This is not paradoxical. *M. capitata* develops verrucae in high flow conditions where high rates of mass transport naturally occur. It is the colony without verrucae that is found in low flow conditions in nature, and hence needs to have enhanced mass transport rates.
